# Effects of modified elastin-collagen matrix on the thermal and mechanical properties of Poly (lactic acid)

**DOI:** 10.1016/j.heliyon.2023.e19598

**Published:** 2023-08-29

**Authors:** Yezihalem Zena, Melakuu Tesfaye, Zelalem Tumssa, Selvakumar Periyasamy

**Affiliations:** Department of Chemical Engineering, Adama Science and Technology University, Adama, Ethiopia

**Keywords:** Biofilms, Crystallinity, Mechanical properties, Modified elastin-collagen matrix, Poly (lactic acid)

## Abstract

Poly (lactic acid) (PLA) has distinctive characteristics, including biodegradability, biocompatibility, thermal process ability, high transparency and good film-forming ability. However, PLA has some poor properties that limit its wide applicability. These properties include a low crystallization rate, poor thermal stability, and high brittleness. The main objective of this research was to investigate the effect of a modified elastin-collagen (m-ELA-COLL) matrix on the properties of PLA. The ELA-COLL matrix was extracted from broiler skin waste and modified by grafting using lactic acid monomer to facilitate compatibility with PLA. The extracted and modified ELA-COLL matrix was investigated using FTIR, and α-helix and β-sheet structures were confirmed in both cases (pre- and post-modifications). Modified elastin-collagen dispersed Poly (lactic acid) (PLA-m-ELA-COLL) blend films were prepared using the solution casting method and characterized using DSC and UTM. The effect of m-ELA-COLL as a nucleating agent resulted in the degree of crystallinity improvement of 58.8% with 10 wt% m-ELA/COLL loading, and the elongation at break was improved by 161.3% for PLA-40%-m-ELA-COLL with a tensile strength of 33.75 MPa. The results obtained revealed that the biofilms can be considered as a good candidate to be studied further in the packaging industry.

## 1 Introduction

Polymers derived from petrochemical sources are not degradable and are difficult to recycle after their end-use applications, such as packaging [1,2]. To solve this aforementioned limitation of petroleum-based polymers, bio-based polymers have been extensively explored as a feasible option to ensure a sustainable green environment by substituting conventional polymers without compromising the important properties required for end-use applications. Biofilms that are derived from biopolymers can be categorized into two; bio plastics derived from biomass such as cellulose, starch, protein, chitosan, etc. Through chemical modification, and bioplastics fabricated from the synthesis of bio based monomers termed synthetic biopolymers such as poly (lactic acid), and polybutylene adipate terephthalate (PBAT) [[3], [4], [5], [6], [7], [8]]. The main reason for these biopolymers to be considered at this high level is due to their abundance, biodegradability, processing ability and some unique properties possessed by the polymers [[9], [10], [11], [12]]. Among the above mentioned bio-based polymers, linear aliphatic thermoplastic Poly (lactic acid) (PLA) has attracted many researchers because of its promising properties as a substitute for conventional polymers. Ring-opening polymerization is one of the most effective ways of producing commercial grade PLA with controlled l-optical isomers [[13], [14], [15], [16]]. PLA has appealing physicochemical properties, including a high elastic modulus and high transparency [17] and has good thermal process ability [18].

However, it has been difficult to expand the application span of this compatible, non-toxic, and biodegradable bioplastic into, packaging, engineering, medical, and other application areas due to its limitations, in thermal stability, crystallization rate and elongation [17]. PLA has low thermal stability, slow crystallization and high brittleness. Thus in addressing those limitations different bio fillers was incorporated with PLA matrix to form blend film and enhance the properties. Those include ammonium salt-modified cellulose nanocrystals [19], coffee grounds [20], keratin [21] and fish gelatin [22]. In the same manner, elastin has been incorporated within PLA matrix and other polymers to generate tissue scaffolds with fibers and extracellular matrix [23].

The main issue in the process of incorporating different materials into the PLA matrix is compatibility, or the stable dispersion of fillers. In most cases, chemical modification of fillers such as grafting and crosslinking is required in order to ensure stable dispersion and improve the compatibility between the PLA matrix and the fillers. In this research work, grafting of lactic acid on the backbone of the elastin-collagen (ELA-COLL) matrix was performed to improve the compatibility of the protein matrix within PLA. Yet assessing the unique properties of these proteins, especially in investigating the synergetic effect of elastin and collagen on the properties of PLA has not been an issue as it should have been.

In this study, Elastin-collagen (ELA-COLL) matrix was selected as a blend film forming biopolymer due to its non-toxicity, degradability and other unique properties that might change the required essential properties of PLA. Furthermore, ELA-COLL is one of the most essential proteins in the living system, mainly in human skin. The successful introduction of this protein matrix into PLA can extend the applicability of the polymer in various biomedical applications. It can also improve the biocompatibility of PLA and facilitate cell growth. This research work is intended to investigate the potential of the ELA-COLL protein matrix for targeting the elongation and crystallization improvement of PLA. In-situ ologomerization of lactic acid was used in the presence of ELA-COLL to modify the protein matrix. The effect of the loading rate of m-ELA-COLL on the crystallization, thermal stability and mechanical properties of PLA has been investigated. The crystallization behavior of PLA-m-ELA-COLL biofilms was evaluated using differential scanning calorimetry (DSC), an optical microscope was used to characterize the dispersion of m-ELA-COLL matrix into PLA and the film's thermal stability and mechanical properties were analyzed using TGA and UTM, respectively.

## 2 Materials and methods

### 2.1 Materials

The materials and chemicals that have been used in this study are stated as follows: commercial grade (Ingeo™ 2003D (NatureWorks)) poly (lactic acid) (PLA) with a melt flow rate of 6 g/10 min; lactic acid (88%, Loba Chemie Pvt. Ltd. India); sodium chloride (Assay 99%); chloroform (99.99%, Fisher Chemicals the Scientific UK); sodium hydroxide (99.8%, LABKEMICAL India); acetone (99.5% Ranchem industry and trading); broiler skin (collected from the local market) and distilled water.

### 2.2 Extraction of ELA-COLL matrix

10% vol. of 1 M NaCl was used to homogenize the broiler's skin after cutting it into small pieces. After 24 h, the treated broiler skin was washed and defatted for 1 h using acetone (30% vol.). 30% vol. of 0.1 N NaOH was used to suspend the dry skin and heated for 15 min With constant shaking in a boiling water bath. After cooling for 24 h, 0.1 N NaOH was used to extract the residue again at 100 °C for 45 min. NaOH-soluble and insoluble constituents were washed multiple times using distilled water from the separatory funnels and dried in an oven at 80 °C [24].

#### 2.2.1 Modification of ELA-COLL matrix by grafting

Different masses (10, 20, and 30 g) of ELL-COLL matrix were dissolved in 100 ml of lactic acid with an ultrasonication-assisted process. After sonication, the dissolved protein matrix was grafted at 120, 150, and 180 °C for 1, 3, 5 and 7 h. The grafting efficiency (GE%) was estimated using equation (1). To calculate the grafting efficiency, filtration was done to separate the grafted and ungrafted ELA-COLL.

(1) $$Grafting\;efficinecy\;\left(GE\%\right)=\frac{W1}{W2}*100$$

where, W_1_ (g) and W_2_ (g) are the weight of modified matrix (i.e., the weight of the matrix after extraction) and the weight of the unmodified elastin-collagen matrix.

### 2.3 PLA-m-ELA-COLL film preparation

PLA/m-ELA-COLL matrix blend films were prepared with different loadings of m-ELA-COLL matrix (1, 3, 5, 10, 20 wt %.) using the solution casting method. The weight percentages of m-ELA-COLL matrix were based on the mass of PLA. Before the addition of m-ELA-COLL matrix, PLA (3 g) was dissolved in 70 ml chloroform and stirred for 1 h. A different weight percentage of m-ELA-COLL matrixes were dissolved in 20 ml of chloroform. Prior to the preparation of the biofilm, the undissolved ELA-COLL matrix, which is the non-grafted part, is filtered. Then added to the PLA solution in different wt% and stirred for an additional 2 h at room temperature, followed by casting on a Petri dish to obtain PLA/m-ELA-COLL biofilm composite. After being solution casted, the solvent was intended to gradually evaporate and the film dried for 48 h. The prepared films were kept in a desiccator [25,26].

### 2.4 Characterization of extracted ELA-COLL and m-ELA-COLL matrix

#### 2.4.1 Functional group analysis (FTIR)

The chemical change between extracted and modified ELA-COLL was investigated using Fourier transferred infrared radiation (Jasco Serial number A013861790 and Model name FTIR-6600 type A) (Japan). The samples were scanned using total reflectance (ATR) mode in the range of 4000 to 400 cm^−1^ with resolution and scanning rates of 4 cm^−1^ and 64, respectively.

### 2.5 Characterization of PLA-m- ELA-COLL films

#### 2.5.1 Thermal analysis

##### 2.5.1.1 Differential scanning calorimeter (DSC*)*

The crystallization and thermal behavior of PLA-m-ELA-COLL blend film was investigated using a differential scanning calorimeter (model: Shimazdu, DSC 60-Japan). ∼8 mg samples were sealed in crucible and scanned in a temperature range of 25–200 °C with heating/cooling cycle at a rate of 10 °C/min under a nitrogen atmosphere. To erase the thermal history, the samples were kept under isothermal conditions at 200 °C for 3 min. Further analysis was performed using the first heating cycle data. The glass transition temperature (Tg), melting temperature (Tm) and heat of fusion (H_m_) were estimated and degree of crystallization (*Xc*) was calculated using equation (2).

(2) Xc(%)=HfHf0*100

where, H_f_ is Heat of fusion of the prepared bio-films (J/kg) and Hf ^O^ is standard heat of fusion of neat PLA (J/kg) which can determine the degree of crystallinity (Xc).

##### 2.5.1.2 Mechanical properties analysis

The samples were conditioned overnight prior to mechanical testing at 20 °C and 65.78% of temperature and humidity, respectively. This test was performed By the Ethiopian Conformity Assessment Enterprise following the ASTM D638 [27,28] tensile strength measuring procedure using the A-5000 Testometric (UK) universal tensile strength testing machine. The force at break, young modulus, and tensile strength were done simultaneously. Additionally, the annealing effect on the mechanical properties of the blend films that were incorporated with a lower (10%) and higher (40%) loading rate of the m-ELA-COLL matrix was analyzed.

## 3 Results and discussion

### 3.1 Functional groups analysis of elastin-collagen (ELA-COLL) matrix

**Fig. 1:**
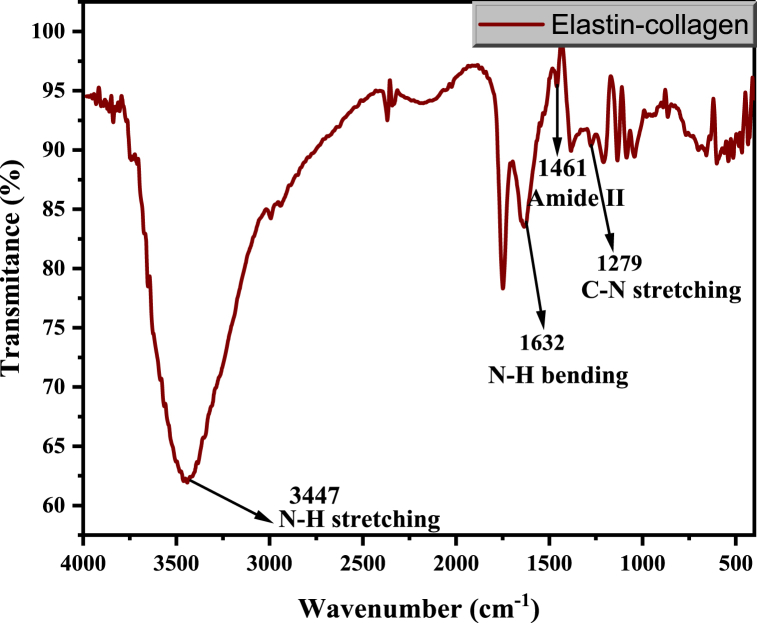
FTIR spectra of ELA-COLL matrix.

The chemical bond of the extracted ELA-COLL was examined using FTIR. The FT-IR spectra shown in Fig. 1 revealed N–H stretching at a wavelength of about 3440 cm^−1^. The isolated ELA-COLL matrix shows the presence of characteristics functional groups for amide I, amide II, and amide III groups. The characteristic absorption peaks for the β sheet structure of elastin matrix [29] and Amide II group [30] are observed at 1632 cm^−1^ and 1461 cm^−1^,respectively. The peak that appeared at 1279 cm^−1^ of the amide III band is attributed to C–N stretching of the ELA-COLL matrix, and all are aligned with previously reported research articles [31]. Additionally, the proline side chain's CH_3_ wagging vibration, which frequently occurs for both collagen and elastin and α-helix of collagen, was indicated by the peak measured at 1383 cm-1 and 978 cm^−1^ [32].

### 3.2 Grafting efficiency of ELA-COLL matrix

The preliminary investigation performed in this work shows poor dispersion of the ELA-COLL matrix in the PLA matrix. A modification of the ELA-COLL is performed in order to improve the dispersion. Grafting was done using lactic acid and a one-variable-at-a-time strategy was used to understand the effect of time, temperature and mixing ratio protein matrix to lactic acid (ELA-COLL/LAC) on grafting efficiency (GE%). The same amount of lactic acid volume was used to dissolve different amounts of the ELA-COLL matrix, which was then grafted at different temperatures and reaction times. All the parameters and their respective grafting efficiencies are shown in Table 1.

**Table 1:** Grafting efficiency of ELA-COLL matrix using lactic acid (LAC).

Sample	Time (h)	Temperature (^o^C)	Mixing ratio (g/100 ml)	Grafting efficiency (%)
ELA-COLL/LAC	1	150	20	50
ELA-COLL/LAC	3	150	20	52
ELA-COLL/LAC	5	150	20	**72.8**
ELA-COLL/LAC	7	150	20	58
ELA-COLL/LAC	5	150	30	44
ELA-COLL/LAC	5	150	10	60
ELA-COLL/LAC	5	120	20	32
ELA-COLL/LAC	5	180	20	52.18

#### 3.2.1 Effect of ELA-COLL mixing ratio on grafting efficiency

The effect of the mixing ratio of ELA-COLL/LAC was examined for the range of 10–30 g of ELA-COLL in 100 ml LAC. As shown in Table 1, the pattern of GE% was increasing, but when more concentration of the matrix was added, it started to decrease. The ELA-COLL/LAC weight ratio of 1:5 had the highest observed GE% of 72.8%. This finding may be explained by the fact that LAC molecules are readily available on the ELA-COLL backbone, allowing for more efficient chain propagation with LAC. As a result, the grafted LAC used up the available active sites of the ELA-COLL protein matrix. On the other hand, the decreasing pattern in grafting efficiency. This is in agreement with the results obtained for the grafting of styrene onto carboxymethyl chitosan [33].

#### 3.2.2 Effect of reaction temperature

While response time and the weight ratio of ELA-COLL/LAC were held constant, grafting was done at various temperatures (120–180 °C). GE% was seen to rise when the temperature rose from 120 to 150 °C. The temperature increment resulted in a reduction of the reaction medium's viscosity, causing more frequent collisions between the reactants and enhanced monomer mobility [34]. However, temperature above 150 °C caused the GE% to decrease, and it may be due to the result of the protein matrix's denaturation. Whereas at lower temperatures the lowest GE% was observed, this might be due to the availability of low activation energy to start up the grafting.

#### 3.2.3 Effect of reaction time

At a constant mixing ratio of ELA-COLL/LAC and reaction temperature, the impact of reaction time was studied. The GE% increased as the reaction time increased from 1 to 5 h. The GE% at 1 and 3 h was observed to be lower, which might be attributed to the incomplete reaction between the ELA-COLL matrix and LAC. The rise in homopolymerization of LAC was caused by the decrease in GE% over 5 h. In general terms, new compatible film-forming materials were successfully prepared by grafting LAC onto the ELA-COLL backbone, which is confirmed by the FTIR analysis. The optimum grafting parameters were found to be 1:5 ELA-COLL/LAC weight ratio, 150 °C, and a 5 h reaction time.

**Fig. 2:**
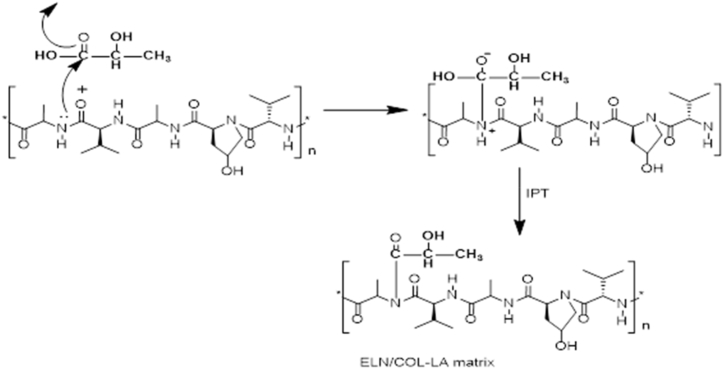
Reaction scheme of grafting of ELA-COLL matrix.

As shown in Fig. 2, the possible reaction mechanism that can occur during the grafting modification process would be internal proton transfer (IPT), in which the amine group of the ELL-COLL matrix would react with the C=O group of lactic acid, resulting in amide formation. And also, this reaction involves poly-condensation; as shown in Fig. 2, the –OH group from lactic acid reacted with the hydrogen from the protein and left the system. Yet, as explained earlier, there was no 100% GE%, which led us to consider the ungrafted parts of the protein matrix.

#### 3.2.4 Functional groups analysis of grafted and ungrafted ELA-COLL

**Fig. 3:**
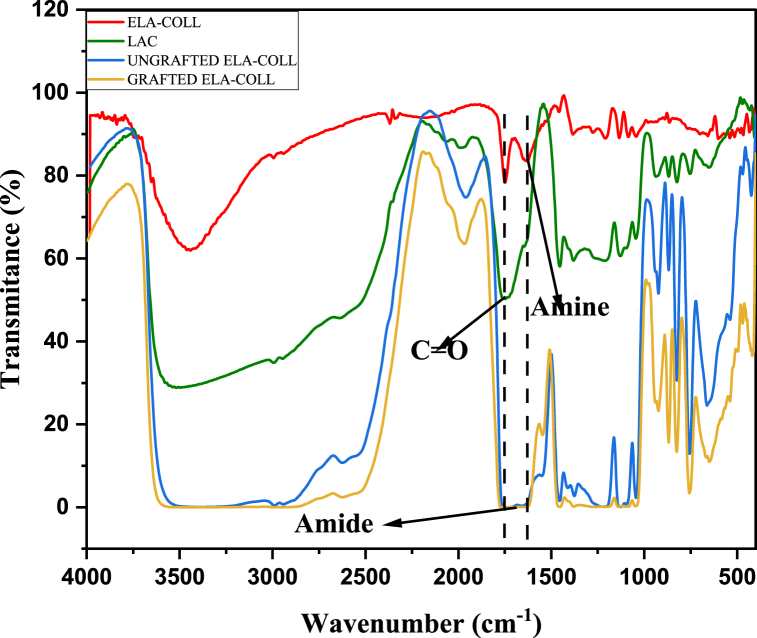
FTIR spectrum of ELA-COLL, LAC, grafted and ungrafted ELA-COLL.

To analyze how the grafting of LAC on the backbone of the ELA-COLL matrix brought a structural and functional change to the pristine ELA-COLL matrix, FTIR analysis of the grafted and ungrafted ELA-COLL matrix was conducted; here, the FTIR of LAC and pristine ELA-COLL were used as references to show how the changes happened in the protein matrix when it was grafted. The FTIR spectra of pristine ELA-COLL, LAC, modified and unmodified ELA-COLL are shown in Fig. 3. The modified and unmodified ELA-COLL matrix showed a broad peak at 3334.34 cm^−1^ due to free OH that came from unreacted lactic acid and NH groups that are formed when the amine group of the ELA-COLL reacted with that of the C=O group of the lactic acid. This phenomenon was reported by Kaith et al. [35], when soy protein grafts copolymerization with ethyl methacrylate.

Whereas the N–H stretching that was found on the pristine ELA-COLL matrix was around 1640 cm^−1^, this specific peak has shown a broad peak when it is modified and shifted to 1692 cm^−1^. This shift in wavenumber and broadening of the peak have proven the amide group formation by the grafting of LAC on the ELA-COLL backbone [31]. Comparing the modified and unmodified systems, the peak broadening of the modified ELA-COLL was higher than that of the unmodified ELA-COLL, which indicates the favored formation of a new amide group. The peak attributed to Amide II in the pristine ELA-COLL matrix was found at 1461 and 1546 cm^−1^, whereas the modified ELA-COLL matrix showed a broadening peak at 1457 cm^−1^, which showed the formation of an amide bond.

In general, the modification process was done and confirmed by the FTIR, in which peak broadening and the formation of new peaks were detected for the modified ELA-COLL matrix, which proved the formation of an amide group. Yet the difference between the grafted and ungrafted ELA-COLL matrix was that the newly formed peaks had a higher intensity in the grafted ELA-COLL matrix than in the ungrafted one.

#### 3.2.5 Image analysis for dispersion of m-ELA-COLL matrix into PLA

**Fig. 4:**
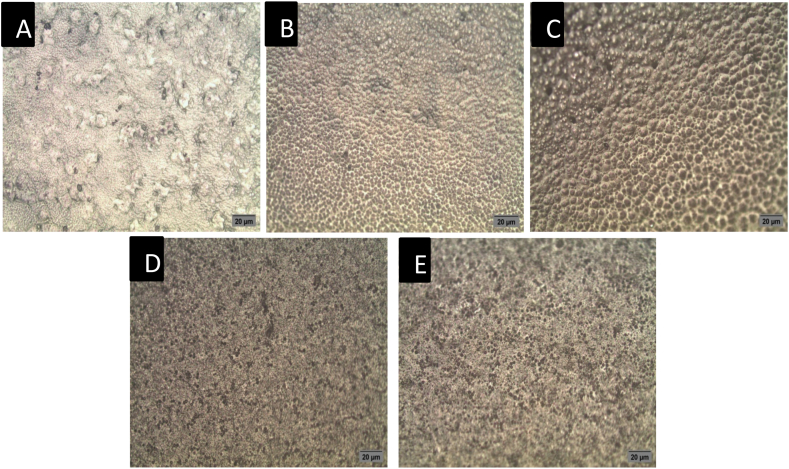
Optical microscope analysis neat PLA (A) and PLA-m-ELL-COLL (10% (B), 20% (C), 30% (D), and 40% (E)) with magnification of 1000×.

The optical microscope image analysis was used to study the dispersion of the ELA-COLL matrix into the PLA matrix. The investigation was done for the PLA-m-ELA-COLL blend with loading rates of 0%, 10%, 20%, 30%, and 40% and all the films were annealed at 110 ^O^C for 150 min. Fig. 4 (A) demonstrated the neat-semi-crystalline PLA's structure. These structures may be more significant than they appear to be due to the influence of the annealing process. The neat-PLA film was used as a baseline in this image analysis to show how the protein matrix diffused and developed some additional structure.

With the addition of 10 wt % of the ELA-COLL matrix, there was a change in macro-level dispersion in PLA, as it can be detected in Fig. 4 (B). The changes that were observed in the films are indicators for the blending between the ELA-COLL matrix and PLA. As the loading of the protein matrix increased, additional honeycomb-like structures (localized proteins) were identified (Fig. 4 (C, D and E)), which might be attributed to the fact that the blending between the PLA and ELA-COLL matrix was not favored as the loading rate of the ELA-COLL matrix increased. These phenomena might lead us to conclude that the blending degree between two polymers is the key factor in providing different properties to the PLA. The formation of honeycomb-like structures on the surface of PLA matrix was due to the high concentration of ELA-COLL matrix which compromises the blending of these two polymers.

#### 3.2.6 Nucleation effect of m-ELA-COLL matrix on crystallization of PLA

**Fig. 5:**
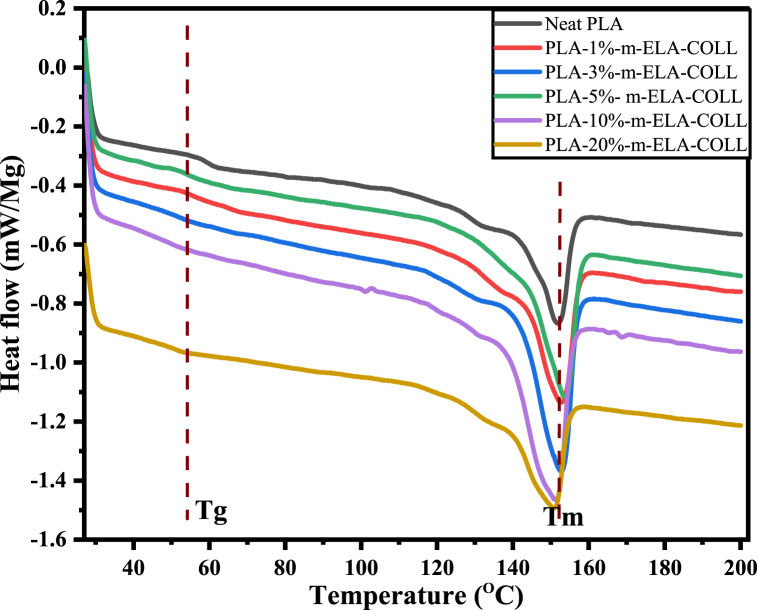
First heating DSC curves at 10 °C/min for neat PLA and PLA-m-ELA-COLL blend biofilms with different loading rate.

Fig. 5 shows the first heating cycle for the crystallization behaviors of the PLA and PLA/m-ELA-COLL films. The effect of different loadings of m-ELA-COLL (1%, 3%, 5%, 10% and 20% wt.%) on the crystallization property of PLA was investigated. Table 2 summarizes the thermal properties of films estimated from the DSC curves. As indicated in Table 2, melting temperature has not been that much affected as m-ELA-COLL content increased. This is due to the occurrence of comparable chain mobility in the crystalline area, which rose from the plasticizing effect of the m-ELA-COLL matrix and also to the increasing entanglements of m-ELA-COLL, which prevented the matrix chains from developing larger crystalline domains. As the loading rate of the m-ELA-COLL matrix increased, there was an increment in H_f_ (J/g), which revealed that the PLA matrix started to fold on the m-ELA-COLL nucleating agent. However, the H_f_ (J/g) of the biofilms at 20% loading of m-ELA-COLL is observed to decrease; this phenomenon confirms that as the loading rate increased, the chain mobility around the crystalline area of PLA film prevented the development of larger crystalline domains [36].

**Table 2:** Glass transition temperature (T_g_), Melting temperature (T_m_), Heat of fusion (H_f_) and calculated degree of Crystallinity of neat PLA and PLA-m-ELL-COLL (1%, 3%, 5%, 10%, 20%).

FILMS	T_g_ (^O^C)	T_m_ (^O^C)	H_f_ (J/g)	X_c_ (%)
Neat PLA	58.7	151.8	30.0	32.0
PLA-1%-m-ELA/COLL	52.0	150.8	35.8	38.2
PLA-3%-m-ELA/COLL	51.2	151.7	43.3	46.3
PLA-5%-m-ELA/COLL	46.5	151.3	47.2	50.6
PLA-10%-m-ELA/COLL	51.2	151.1	48.0	51.3
PLA-20%-m-ELA/COLL	46.2	150.7	35.8	36.9

The T_g_ from DSC analysis started to fall between 58.8 ^O^C and 51.9 ^O^C when the m-ELA-COLL matrix content rose from 0 wt% to 1 wt%, which can be attributed to the matrix causing a decrease in the entanglement nature of PLA. In other words, the interaction between m-ELA-COLL and PLA molecular chains takes the place of the interaction that occurs within the original PLA molecular chains and weakens the molecular chain segments interaction force. This was the reason why the blend films exhibits a slightly lower glass transition temperature than that of pure PLA [37,38] and it might also be due to the plasticizing effect due to the chain flexibility of the m-ELA-COLL matrix that protrudes in the PLA phase [39].

The major scientific fact behind this shift is that when PLA and m-ELA-COLL are dissolved in a solvent, the m-ELA-COLL will lose its secondary structures, and the entangled PLAs will somehow separate and forms a number of single chains. Due to this, the interactions might be changed to PLA-m-ELA-COLL-PLA instead of PLA-PLA, which is surrounded by solvents. When the blend film was casted, the solvent will evaporate through slow drying. Throughout the slow drying process, there was the formation of a one-phase system that would support the idea of the favored dispersion of m-ELA-COLL in the PLA matrix. PLA started to fold on the surface of the m-ELA-COLL matrix when the films were exposed to annealing, and here the crystallization process started. However, the m-ELA-COLL matrixes have synergetic properties that would make them serve as plasticizers and r as nucleating agents. As the loading of m-ELA-COLL increases the interaction between PLA and m-ELA-COLL starts to be compromised and m-ELA-COLL/m-ELA-COLL interaction will be favored due to the formation of localized protein aggregates.

#### 3.2.7 Thermal stability of PLA-m-ELA-COLL bio-films

**Fig. 6:**
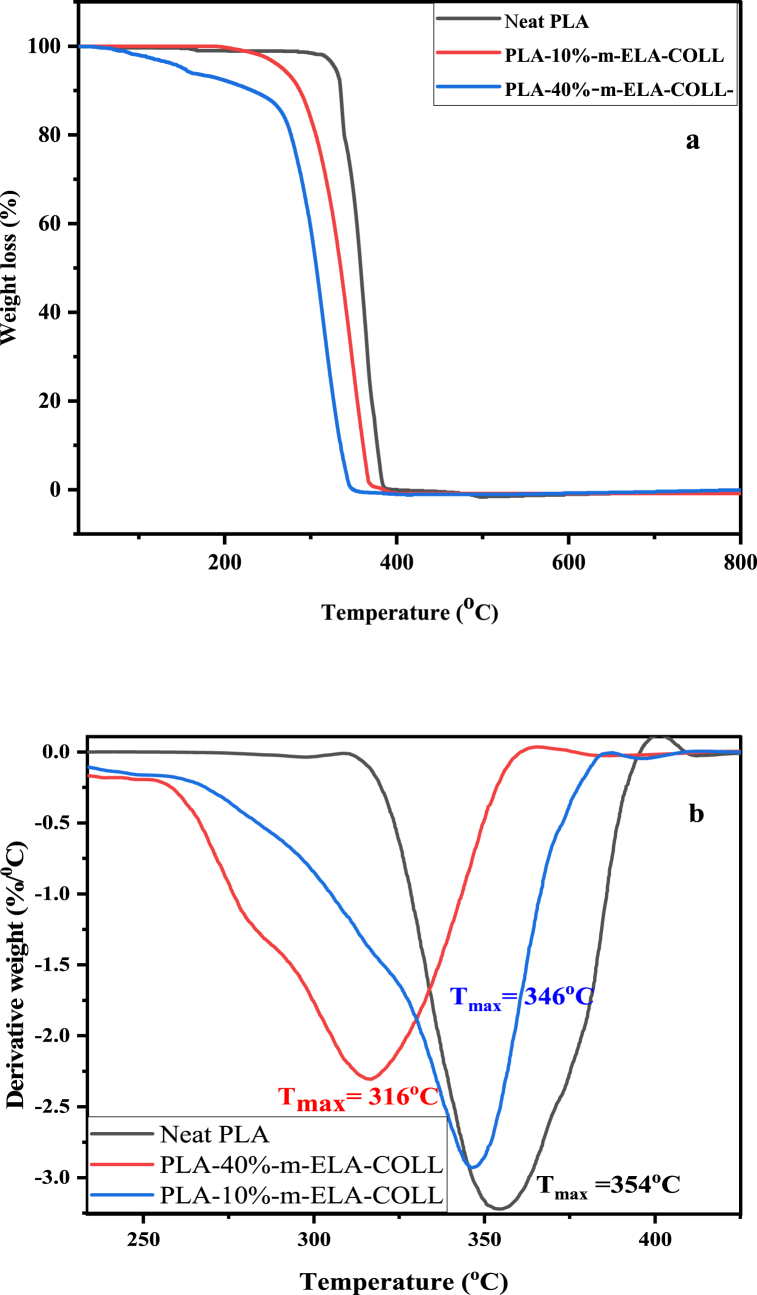
(a) TGA and (b) DTG curves for PLA, PLA-10%-m-ELA-COLL and PLA-40%-m-ELA-COLL.

The effect of m-ELA-COLL on the thermal stability of PLA-m-ELA-COLL blend films was investigated using Thermogravimetric Analysis (TGA). The TGA and DTG curves of neat PLA, PLA-10%-m-ELA-COLL and PLA-40%-m-ELA-COLL are shown in Fig. 6 (a and b). The degradation of PLA started at a lower temperature when incorporated with a different loading rate of the m-ELA-COLL matrix. As shown in Table 3, the T_10%_ and T_90%_ decreased proportionally with the increase in m-ELA-COLL percentages, which suggests that m-ELA-COLL accelerated the thermal degradation of PLA polymers due to its lower thermal resistance property.

**Table 3:** Thermal characteristics of neat PLA, PLA-10%-m-ELA-COLL and PLA-40%m-ELA-COLL.

Films	T_10_ (^o^C)	T_90_ (^o^C)	T_max_ (^o^C)
Neat PLA	336	377.4	354
PLA-10%-m-ELA-COLL	283	361.4	346
PLA-40%-m-ELA-COLL	233	334.6	316

The Addition of 40%-m-ELA-COLL to the PLA matrix shifted the thermal decomposition of PLA-m-ELA-COLL blend films to a lower temperature Fig. 6 (a). This decreasing effect on the thermal stability of the PLA-based films might be explained by the fact that the introduction of the m-ELA-COLL matrix into PLA created loosely entangled PLA matrixes that had higher thermal stability. Other studies with comparable findings have been published [40]. The TGA curve showed a consistent and smooth degradation process after 200 °C and these findings suggest m-ELA-COLL and PLA in the composites have good thermodynamic compatibility.

As indicated in Fig. 6 (b), with the addition of m-ELA-COLL, the maximum degradation temperature tends to be lower, from 354 °C for neat PLA to 316 °C for PLA-40%-m-ELA-COLL. This reduction might be due to the plasticization effect of m-ELA-COLL. This kind of reduction in Tmax occurred when starch acetate was incorporated with PLA [41]. The largest weight loss (T_max_) for all mix bio-nanocomposites was seen between 316 and 354 °C, which is also the temperature range at which PLA and m-ELA-COLL degraded the most. The blend system has one distinct maximum decomposition rate peak, which explains the preferred compatibility between the m-ELA-COLL and PLA.

#### 3.2.8 Mechanical properties of PLA/m-ELA-COLL films

Mechanical testing was done to see how the addition of m-ELA-COLL affected the mechanical properties of the PLA. Table 4 displays the mechanical characteristics of PLA-m-ELA-COLL biofilms made of PLA at four levels of 0%, 10%, 20%, 30%, and 40%. For mechanical characteristics like tensile strength, elongation at break, and Young's modulus, sample values were taken into account.

**Table 4:** Elongation at break, tensile strength, and young's modulus of neat PLA and PLA/m-ELL-COLL blend biofilm with different loading without annealing.

Sample name	Elongation at break (%)	Change in Elongation at break as compare to NPLA (%)	Young's Modulus (N/mm^2)^	Stress at break (N/mm^2^)
Neat PLA	3.1 ± 0.3	–	3757.4 ± 133	63.5 ± 2.7
PLA-10%-m-ELL-COLL	4.2 ± 0.4	35.5	3583.5 ± 35	60.9 ± 1.3
PLA-20%-m-ELL-COLL	5.4 ± 0.4	74.2	2507.5 ± 27.2	34.7 ± 1.1
PLA-30%-m-ELL-COLL	6.4 ± 0.6	106.5	2413.4 ± 66	31.3 ± 1
PLA-40%-m-ELL-COLL	8.1 ± 0.8	161.3	2133.7 ± 59	28.7 ± 1.1

Generally, it can be concluded that the protein matrix favored the elongation at the break point of biofilm; on the contrary, the tensile strength and young's modulus decreased as the loading rate of the protein matrix increased.

**Fig. 7:**
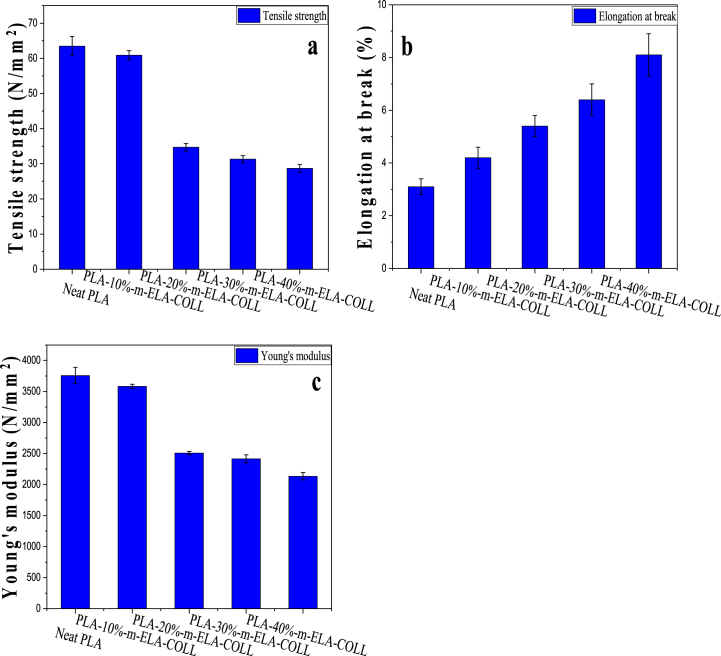
The effect of m-ELA-COLL loading on (a) Tensile Strength (b) Elongation at break point (c) Youngs modoulus of PLA-m-ELA-COLL with annealing.

The tensile strength (TS) is depicted in Fig. 7(a). According to the graph, PLA films with a 30% *m*-ELA/COLL matrix have a tensile strength of 32.25 N/m, whereas pure PLA film has a tensile strength of 67 N/m. As can be seen from the graph, with the incorporation of *m*-ELA-COLL matrix with PLA, the tensile strength of the biofilms is lower than that of PLA, which may be attributed to the plasticizing effect of protein matrixes on the film. It causes a drop in tensile strength as protein matrix loading increases. This trend remained the same until the loading reached 40%. Beyond that, the trend shifted to an increasing pattern, yet the increment was not significant. For maximum loading of the protein matrix, the TS significantly decreased up to 54.8% compared to neat PLA. Díez-Pascual et al. (2014) [36,42,43] stated that the degree of crystallization can be directly related to the tensile strength of particular biofilms. Comparing the blend samples alone, the tensile strength of the PLA films with a 10% m-ELA/COLL matrix was higher. For this phenomenon, the possible reasons behind it are the highest degree of crystallinity and the plasticizing effect of m-ELA-COLL as the loading increased. These phenomena can also be correlated with DSC analysis.

The blend films with different protein loading rates (10%–40%) showed a significant improvement in elongation at break, as shown in Fig. 7(b), which ranged from 35.5% for PLA-10%-m-ELA-COLL to 161.3% for PLA-40%-m-ELA-COLL as compared to the elongation at break obtained for neat PLA. As the rate at which the protein matrix loading increased, the elongation at the break also increased. This could be due to the plasticizing effect of the m-ELA-COLL matrix, which facilitates chain mobility. According to this, secondary bonds might be formed between the amino groups of m-EAL-COLL and the OH groups of PLA.

From the mechanical property analysis, the young's modulus reduced by 4.6% when 10% of the m-ELA-COLL matrix was incorporated within the PLA matrix, as shown in Fig. 7(c). As the loading rate of the m-ELA-COLL matrix increases, there is a significant reduction in young's modulus. The significant fall in modulus value at 20–40 wt% m-ELA-COLL loading explained the possibility of agglomerations of localized protein molecules, which reduce the intermolecular mixing between PLA and m-ELA-COLL matrix and entanglements of m-ELA-COLL at higher loading [36].

##### 3.2.8.1 Effect of annealing temperature on the mechanical property of PLA/m-ELA-COLL films

**Fig. 8:**
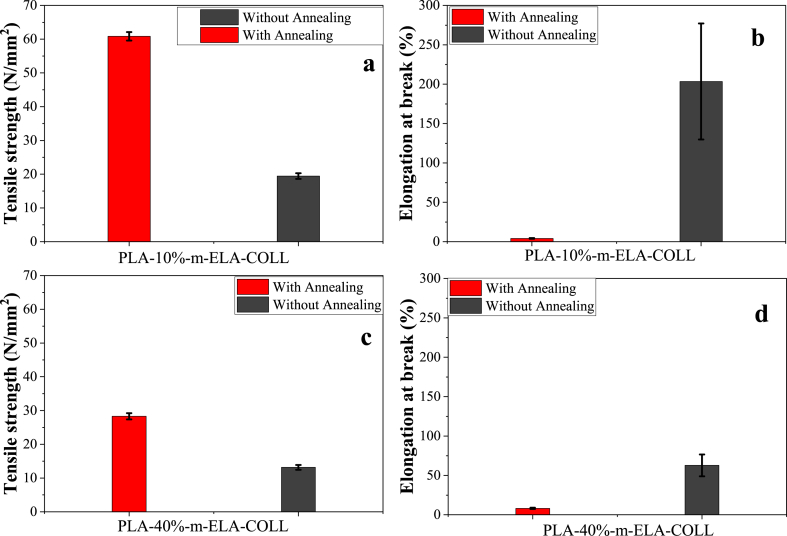
The effect of annealing on the Tensile strength and Elongation at break for PLA-10%-m-ELA-COLL (a and b) and PLA-40%-m-ELA-COLL (c and d) respectively.

The effect of annealing temperature on the mechanical properties of m-ELA-COLL dispersed PLA biofilms was studied in terms of how the biofilm's mechanical properties were affected and what would be the possible reason behind these phenomena. As it is shown below in Fig. 8 (b), the elongation at break for PLA-10%-m-ELA-COLL films was higher for the film analyzed under no thermal effect (without annealing). This might be due to the fact that the intermolecular interaction between m-ELA-COLL and PLA might be favored, i.e., no localized protein was there that halted the effect of the m-ELA-COLL matrix, which facilitated the elasticity properties of m-ELA-COLL on the PLA. Whereas the other possibility might be that since there was no thermal effect, the nucleation effect of m-ELA-COLL was not facilitated; this nucleation effect is the main factor in enhancing tensile strength but here, since there was no effect of annealing, tensile strength was lower than that of elongation at break.

On contrary in case of the tensile strength of the biofilms, it was favored when the film was annealed; this might be due to the favored nucleation effect of the m-ELA-COLL matrix on the PLA Fig. 8 (a). These phenomena were supported with a high degree of crystallinity, while the film that was not annealed showed lower tensile strength. An increase in chain mobility upon annealing and the presence of stable nucleation sites facilitated crystallization, which leads to higher tensile strength [44,45].

In the same manner, the effect of annealing temperature on the PLA/40%-m-ELA-COLL biofilms was studied, which resulted in higher elongation at break and higher stress at the break was observed for the biofilm that was annealed. As it is shown in Fig. 8 (d), the elongation at break of annealed PLA-40%-m-ELA-COLL biofilm was favored over that of the un-annealed PLA-40%-m-ELA-COLL bio-films, this might be due to the localized proteins going through some thermal relaxation due to the effect of temperature and a plasticizer. In the case of tensile strength (Fig. 8 (c)), the effect of annealing temperature is always related to a higher degree of crystallinity. As the films passed through some thermal processes the nucleation effect of the m-ELA-COLL might be favored, this resulting in higher tensile strength.

## 4 Conclusions

Extra-cellular proteins, which are found in broiler skin, were extracted and modified through grafting using lactic acid (LA). In order to analyze the formation of the ELA-COLL matrix and how it was modified, FTIR analysis was performed. The analysis results revealed that the formation of ELA-COLL matrix was confirmed by the Amide I, II and III characteristic peaks. While dealing with the modified and unmodified ELA-COLL matrix, the formation of broad peaks has proved that the poly-condensation reaction resulted in the formation of an amide bond. For PLA/m-ELA-COLL biofilms, the crystallization effect of the m-ELA-COLL matrix was studied and resulted in 51.3% degree of crystallinity with a 10% wt. Loading rate, whereas the elongation at break point was improved by 143.1% with a 40% wt. Loading rate compared to neat PLA. For the case of tensile strength, a 54.8% decrement was observed for the highest loading rate of m-ELA-COLL, and the young's modulus also exhibited a decrement pattern.

## Author contribution statement

Melakuu Tesfaye: Conceived and designed the experiments; Analyzed and interpreted the data; Contributed reagents, materials, analysis tools or data; Wrote the paper. Yezialem Zena: Conceived and designed the experiments; Performed the experiments; Analyzed and interpreted the data; Wrote the paper. Zelalem Tumssa: Conceived and designed the experiments; Analyzed and interpreted the data; Contributed reagents, materials, analysis tools or data. Selva Kumar: Analyzed and interpreted the data; Contributed reagents, materials, analysis tools or data.

## Data availability statement

Data included in article/supplementary material/referenced in article.

## Declaration of competing interest

The authors declare that they have no known competing financial interests or personal relationships that could have appeared to influence the work reported in this paper.
